# Interferon (IFN) and Cellular Immune Response Evoked in RNA-Pattern Sensing During Infection with Hepatitis C Virus (HCV)

**DOI:** 10.3390/s151027160

**Published:** 2015-10-23

**Authors:** Masato Nakai, Hiroyuki Oshiumi, Kenji Funami, Masaaki Okamoto, Misako Matsumoto, Tsukasa Seya, Naoya Sakamoto

**Affiliations:** 1 Department of Microbiology and Immunology, Graduate School of Medicine, Hokkaido University, Kita-ku, Sapporo 060-8638, Japan; E-Mails: oshiumi@czc.hokudai.ac.jp (H.O.); kfunami@med.hokudai.ac.jp (K.F.); okamoto@med.hokudai.ac.jp (M.O.); matumoto@pop.med.hokudai.ac.jp (M.M.); 2 Department of Gastroenterology, Graduate School of Medicine, Hokkaido University, Kita-ku, Sapporo 060-8638, Japan; E-Mail: sakamoto@med.hokudai.ac.jp

**Keywords:** Hepatitis C virus (HCV), interferon (IFN)-lambda, dendritic cells (DCs), Toll-like receptor 3 (TLR3), natural killer (NK) cell, cytotoxic T lymphocyte (CTL)

## Abstract

Hepatitis C virus (HCV) infects hepatocytes but not dendritic cells (DCs), but DCs effectively mature in response to HCV-infected hepatocytes. Using gene-disrupted mice and hydrodynamic injection strategy, we found the MAVS pathway to be crucial for induction of type III interferons (IFNs) in response to HCV in mouse. Human hepatocytes barely express TLR3 under non-infectious states, but frequently express it in HCV infection. Type I and III IFNs are induced upon stimulation with polyI:C, an analog of double-stranded (ds)RNA. Activation of TLR3 and the TICAM-1 pathway, followed by DC-mediated activation of cellular immunity, is augmented during exposure to viral RNA. Although type III IFNs are released from replication-competent human hepatocytes, DC-mediated CTL proliferation and NK cell activation hardly occur in response to the released type III IFNs. Yet, type I IFNs and HCV-infected hepatocytes can induce maturation of DCs in either human or mouse origin. In addition, mouse CD8+ DCs mature in response to HCV-infected hepatocytes unless the TLR3/TICAM-1 pathway is blocked. We found the exosomes containing HCV RNA in the supernatant of the HCV-infected hepatocytes act as a source of TLR3-mediated DC maturation. Here we summarize our view on the mechanism by which DCs mature to induce NK and CTL in a status of HCV infection.

## 1. Introduction

Dendritic cell (DC) maturation is a typical phenotype in virus-infected hosts [1]. Virus antigens (Ags) are efficiently presented on the MHC class I in antigen-presenting DCs. Viruses have pattern molecules characteristic to viral products and their genomes, and these patterns stimulate pattern-recognition receptors (PRRs) to up-regulate MHC class I, co-stimulators, as well as cytokine production in DCs [1,2]. Exogenous Ags and pattern molecules are taken up into DCs and PRR-primed DCs present Ag peptides on the MHC via cross-presentation. Viral genome RNAs and their replication products have unique structures that can be discriminated from those of host RNAs, and then act as PRR agonists [3,4]. DCs possess RNA sensors in the cytoplasm and endosome, which recognize different structural motifs of RNAs [2]. RIG-I and MDA5 sense 5′-phosphated- or long-(>1 kb) dsRNA motif, respectively, in cytoplasm, while TLR3 and TLR7 sense stem-structured or single-stranded RNA in endosome, respectively [2,3,4]. DC’s Ag-presenting ability is somehow facilitated in response to viral RNA products, and notably, Ag-presenting DCs (CD141+ DCs in human) express TLR3 and RIG-I/MDA5 [5].

Hepatitis C virus (HCV) is a positive-stranded RNA virus, and selectively infects hepatocytes. Non-infected hepatocytes express only subtle RIG-I/MDA5 and virtually no TLR3, while these sensors are up-regulated in hepatocytes in response to HCV RNA [6,7]. HCV-infected hepatocytes liberate type I and type III interferons (IFNs), as well as cytokines/chemokines [6,7,8]. On the other hand, DCs also induce type I and III IFNs in association with or during co-culture with infected hepatocytes [5,8]. Although HCV does not directly infect DCs [6,9,10], HCV particles are internalized into DCs to be a source of the HCV RNA that stimulates RNA sensors [10]. In HCV infection, which of the two pathways, RIG-I/MDA5 or TLR3, is mainly responsible for initial production of IFNs and particularly involved in IFN-lambda induction remained undetermined [6].

We have examined the role of the intrinsic *vs.* extrinsic pathways in HCV RNA response in mice using hydrodynamic and KO mice models [8]. Extrinsic administration of a dsRNA analog polyI:C brought us a similar result to the HCV RNA response. Our data suggested that the TLR3/TICAM-1/IRF3 pathway is crucial for DCs to mature for cross-priming of T cells and activation of NK cells while IFN-lambda induction barely participates in this process [8], and that an unidentified pathway for viral RNA uptake and delivering to endosome initiates the activation of DCs to license activation of cellular immunity coping against secondary virus infections. Here we summarize our view on how DC maturation is induced by HCV RNA, which constitutes a part of host anti-HCV immune response.

## 2. Hydrodynamic Injection of HCV RNA in Mice

Hepatitis C virus (HCV) infects only a limited number of hosts, such as humans, chimpanzees and tree shrews [11,12]. Neither experimental animal models in mice and rats nor good cell culture systems for HCV (other than JFH1 strain [13]) have been established for testing clinical HCV samples [6], although new models are being developed to overcome this barrier [12]. The presence of this major barrier has disturbed the *in vivo* analysis of HCV and studies on host pattern recognition response to HCV-RNA.

Thus, alternative methods using viral vectors (retrovirus, adenovirus, herpes virus *etc.*) with the HCV genome, have been employed to mimic HCV infection, using gene transfer of plasmid or foreign DNA (exogenous gene) to experimental animals [14]. Hydrodynamic injection is another method for transfer of the viral genome into mice, particularly in the liver. This method was first reported by Liu *et al.* [15], who stated that HCV plasmid is intravenously (i.v.) transfected quickly with large amounts of solution into mouse, which facilitates efficient gene transfer in the liver. A usual condition is rapid injection (5–7 s) of 8%–10% per weight of DNA solution from the tail vein. At this condition, cDNA encoding the HCV genes is transfected into systemic organs, including the liver, lung, heart, spleen, and kidney. In particular, the HCV-encoding DNA accumulates in the liver: gene transfer efficiency of about 30%–40% is attained when 10 μg of plasmid DNA is injected in mice. Hence, this method has been applied to an experimental model for HCV infection. At the beginning, plasmid DNA was utilized for this method [15], and then, hydrodynamic injection of HCV-RNA was tried by Gale *et al*. in 2008 [16]. According to their report, in the HCV-RNA gene transfer using the hydrodynamic injection, expression of type I IFN is observed depending on the RIG-I pathway. The method therefore is a specific way for HCV-RNA to be efficiently delivered to cytoplasm of the hepatocytes. This gene transfer method has been followed by several further studies [17,18]. The method of hydrodynamic injection was developed not only for HCV-RNA itself [16] but also for other HCV genes [17,18]. Since mice have no inherent HCV infectivity, it is considered to be useful as a way to mimic the HCV infection.

## 3. Extrinsically Transfected RNA Stimulates the MAVS Pathway

There are many nucleic acid sensors in the cytoplasm and the endosome (Table 1). The cytoplasmic RNA sensors RIG-I and MDA5 preferentially recognize RNA transfected by lipofection or hydrodynamic injection, like viral infection [19,20,21]. RIG-I and MDA5, are categorized into RLRs (RIG-I-like receptors), which possess a Caspase Activation and Recruitment Domain (CARD) at the N-terminus, DExD/H box type RNA helicase domain in the center, and a unique C-terminal domain (CTD), which binds and recognizes viral RNA [3,21]. The CARD-like domain at the N-terminus in RIG-I/MDA5 is essential for binding to MAVS, an adapter molecule. RIG-I recognizes a characteristic pattern in the viral RNA for HCV, and through its CARD domain transmits activation signal to MAVS present on the outer mitochondrial membrane, which in turn activates the IRF3/7-activating kinases and then induces activation of IFNα/β and IFN-inducible genes [6,22].

**Figure 1:**
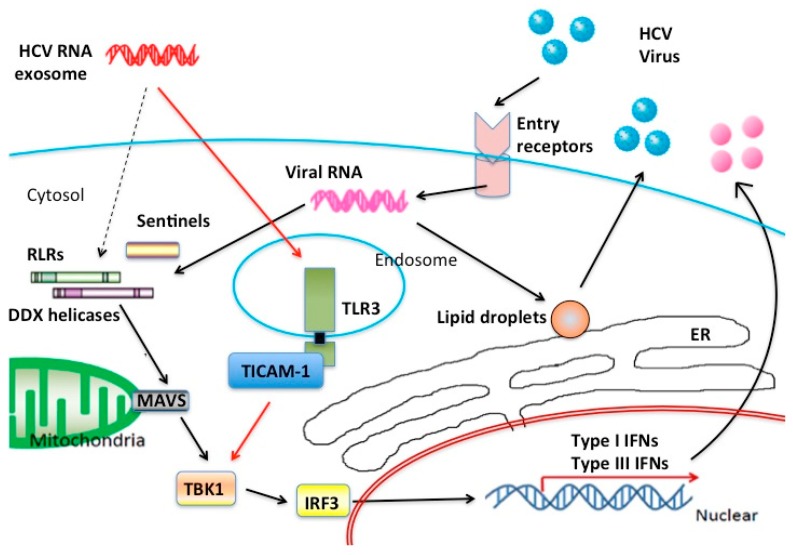
Nucleic acid sensors in HCV infection. Possible innate RNA-sensors participating in the recognition of HCV RNA are illustrated in this figure. In hepatocyte cytoplasm, major RNA sensors are RLR family proteins although many sentinels help RLRs to recognize viral RNA. On the other hand, in stromal cells TLR3 can work as a main sensor for HCV RNA released from the foci of HCV infection. Type III IFNs are the main product of the TLR3 signal in response to exogenous dsRNA. CD8+ DC rapidly secrete IFN-lambda in acute phase HCV infection.

When the HCV causes acute infection in humans, HCV is transported to liver through the blood to infect the liver cells. Some types of B cells may carry HCV as a reservoir to the liver [23,24]. In acute HCV infection in hepatocytes, cell surface molecules such as CD81, SRBI, Occludin, and Claudin-1 serves as HCV receptors [25,26,27,28]. HCV particles are taken up by endocytosis into endosome of the hepatocytes, and HCV RNAs are released from the endosome to the cytoplasm. How the encapsidated viral RNAs are liberated from the endosome to the cytoplasm remains unknown. Anyhow, viral RNAs are replicated via an intermediate product, dsRNA, which are often externalized to extra-cellular fluid as exosome or cell debris [6,8,10]. Intercellular transfer of HCV-RNA is achieved through this exosome-mediated process besides infection. Considering the process of HCV replication, it is believed that HCV-RNA is recognized by RIG-I in cytoplasm, which causes the production of type I IFN through activation of the MAVS pathway (Figure 1). 

**Table 1:** Nucleic acid sensors involved in IFN-inducing innate immune response.

Pattern-Recognition Receptor	Adaptor	Natural Agonist	Synthtic Agonist	Pathogen
TLR3	TICAM-1	Endosomal dsRNA	poly(I:C)	DNA/RNA virus
TLR7/8	MyD88	Endosomal ssRNA	Imidazoquinoline	RNA virus, bacteria, fungi
TLR9	MyD88	Non-methylated CpG DNA	CpG ODNs	DNA virus, bacteria
RIG-I	MAVS	Cytosolic 5'-ppp-dsRNA	short poly(I:C)	RNA virus, DNA virus
MDA5	MAVS	Cytosolic long dsRNA	long poly(I:C)	RNA virus, bacteria
NOD2	MAVS	Cytosolic ssRNA	muramyl dipeptide	RNA virus, bacteria
DDX3	MAVS	Cytosolic ssRNA, dsRNA	poly(I:C)	RNA virus
DDX1/21, DHX36	TICAM-1	Cytosolic dsRNA	poly(I:C)	RNA virus
DDX41	STING	Cytosolic dsDNA	-	DNA virus, bacteria
DDX60	MAVS	Cytosolic RNA, dsDNA,	poly(I:C)	DNA/RNA virus
DHX9/DHX36	MyD88 (MAVS)	Cytosolic dsDNA (dsRNA)	CpG ODNs (poly(I:C)	DNA virus
DAI (ZBP1)	STING?	Cytosolic dsDNA	-	Dna virus, bacteria
NLRP3	ASC	Cytosolic RNA	sillica, asbestos, alum	DNA/RNA virus, bacteria
IFI16	STING	Cytosolic dsDNA	-	DNA virus
LRRFIP1	β-Catenin	Cytosolic dsDNA	-	DNA virus, bacteria

However, only a little production of type I IFN can be detected in infection experiments using primary hepatocytes or hepatoma cell lines, and *in vivo* studies using chimpanzee [29,30]. This is probably due to the cleavage of MAVS or an ubiquitination enzyme Riplet by NS3/4A protease [31,32]. In fact, evidence is shown that ubiquitination of RIG-I by Riplet is indispensable for MAVS activation [33]. Type I IFN production via the RIG-I/MAVS pathway is therefore suppressed by the proteolytic cleavage of Riplet. On the other hand, experiments using human hepatocyte lines, HepG2, Huh7 and primary hepatocytes, and *in vivo* studies with chimpanzee, rapid induction of Type III IFN is a characteristic feature in HCV infection [29,30]. Based on these findings, a protagonist of the initial response to HCV would be type III IFN rather than type I IFN. Type III IFN can suppress HCV replication in experiments with HCVcc or replicons [34,35]. In these cases, type III IFN actually expresses anti-HCV activity [36]. Type III IFN response is inhibited in HCV-infected cells [37], where MAVS in peroxisomes plays a critical role in the induction of type III IFNs [38]. It is presumed that HCV NS3/4A protease localized to the peroxisomes inactivates MAVS [39], resulting in the suppression of type III IFN induction. Hence, NS3/4A protease may suppress not only type I IFN but also type III IFN [40]. Phase I study with PEG-IFN-lambda has been performed [41], and so far the report reflects high safety and significant decrease of HCV-RNA by PEG-IFN-lambda as compared with PEG-IFN-α. Future reports are awaited with respect to its clinical efficacy.

Because HCV is a hepatotropic virus, it primarily infects and proliferates in hepatocytes. The possibility of infection in non-parenchymal cells is being debated, sometimes with controversial views [42,43]. There are significantly high frequencies of mixed cryoglobulinemia and B cell malignant lymphoma in HCV patients [44], suggesting that HCV infects some blood cells, including B cells [23,44]. However, HCV infection to dendritic cells has been negated in several reports [10,42].

Type I and III IFNs are produced through the RIG-I/MAVS pathway in hepatocytes when HCV infects hepatocytes as described in previous papers [6,30,31,45]. Recently, many sentinels, belonging to DEAD box-containing helicases, help RIG-I/MAVS to recognize viral RNAs. In the context of RNA recognition, ubiquitin E3 ligases TRIM25 and Riplet participate in RIG-I ubiquitination that allows RIG-I to change to an active conformation to facilitate the access of RNA to RIG-I [46]. We found that hydrodynamic injection of HCV-RNA (ssRNA, dsRNA) or gene transfer of RNA harvested from O cells (that contains HCV replicon) into mice resulted in upregulation of not only type I but also type III IFN in the liver and blood serum [8]. These results may support the *in vitro* data on the liver cell lines [29,30] and *in vivo* chimpanzee studies [30].

On the other hand, hepato-parenchymal cells exist with non-parenchymal cells consisting of fibroblasts, hepatic stellate cells, macrophages including Kupffer cells, and dendritic cells. Type III IFN production may be originated from the non-parenchymal cells or immune cells other than liver parenchymal cells in *in vivo* HCV infection or transfection of HCV-RNA by hydrodynamic injection. Therefore, we verified the *ex vivo* IFN production in CD8+ DCs that were separated from the spleen of mice that had been treated with HCV-RNA or HCV replicon by the hydrodynamic injection [8]. We found type III IFN was rapidly produced in CD8+ DCs in a MAVS-dependent manner.

Although HCV JFH1-strain fails to infect DCs *in vitro* [10], previous studies indicate that HCV infects DCs in chronically infected patients [47,48]. If human patients’ DCs could be infected with HCV as well as hepatocytes, the MAVS pathway would play a pivotal role in type III IFN production. This point leads us to think the source of Type III IFN. Exosomes containing HCV RNA can be released from HCV-infected hepatocytes [10,49,50,51], by which HCV RNA might be delivered from the hepatocyte cytoplasm to endosomes of dendritic cells. Type III IFN induction occurs in CD8+ DC via the TLR3/TICAM-1 pathway [8]. Artificial transfection of HCV-derived RNA using hydrodynamic injection reflects temporal *in vivo* dynamics of HCV RNA but not actual chronic infection where HCV RNAs are persistently released. MAVS-dependent Type I/Type III IFN production by hydrodynamic injection may not necessarily reflect the event occurring in HCV infection in humans.

When CD8+ DCs are co-cultured with HCV replicon-positive cells *in vitro*, type I and III IFNs are shortly produced in the supernatant [8]. If CD8+ DCs from MAVS knockout or TICAM-1 knockout mice are used for the co-culture study, one can find this phenomenon as MAVS-independent, but TICAM-1-dependent. This mechanism is not always elucidated, but most possible is that through the exosome containing HCV-RNA, RNAs are transferred from HCV-infected liver cells to DCs [50]. If this is the case, intercellular transfer of HCV RNA occurs for DC maturation without an infectious process [8,50]. On the other hand, other reports indicate that cell-to-cell contact between HCVcc-producing hepatocytes and human BDCA3+ DCs enables DCs to produce type III IFN [52]. If BDCA3+ DC acts as a counterpart of the CD8+ DC of mice in viral infections [5], cell-cell contact in infectious millieu may allow DCs to produce type III IFN and mature for induction of immune effectors [52]. The level of type III IFN production is unchanged even when HCV replication is inactivated with UV in mice [52], suggesting that CD8+ DC is necessary to produce type III IFN through taking exogenous HCV-RNA to DCs for TLR3 activation. BDCA3+ DC (a CD8+ DCs counterpart) is a major source of type III IFN production in humans HCV infection; however, it is not clear whether this mechanism is MAVS-dependent or TLR-3/TICAM-dependent in human.

## 4. The IFN-Lambda Receptor in CD8+ DC Fails to Activate Cross-Priming or NK Activation

IFN-lambda receptor consists of two components of IFN-LR1 (IL28-Rα) and IL-10R2 [53,54]. Generally, IL10R2 is expressed ubiquitously in many human cells, but IFN-LR1 is expressed predominantly in epithelial cells. IFN-LR1 determines the response to IFN-lambda in a cellular level in human [55]. So, we tested if this is true in mouse DCs. The results are that among DC subsets tested in our mouse study, the double-negative DCs and CD4 + DCs do not express IFN-LR1 (IL28-Rα) but CD8+ DC express it [8].

When CD8+ DCs are stimulated with type I IFN, co-stimulators such as CD40, CD80 and CD86, are up-regulated on the cell surface [56,57]. Thus, they are activation markers of DCs. Enhanced expression of these activation markers is not observed in stimulation with type III IFN [8]. However, it is unlikely that CD8+ DCs are activated in response to type III IFN in a feed-back loop [8]. In addition to cross-priming ability, CD8+ DCs generally have high ability of NK activation [58]. DCs express NK-activating ligands for NK activation by recognizing a dsRNA, PolyI:C [10,59]. In addition, the exogenous type I IFN induces cross-priming for T cell proliferation in CD8+ DCs [60]. However, in our study, CD8+ DCs do not induce efficient cross-presentation or NK activation in response to type III IFN, unlike the case of type I IFN (monitored by enhanced expression of CD69 and IFN-γ production) [8].

**Figure 2:**
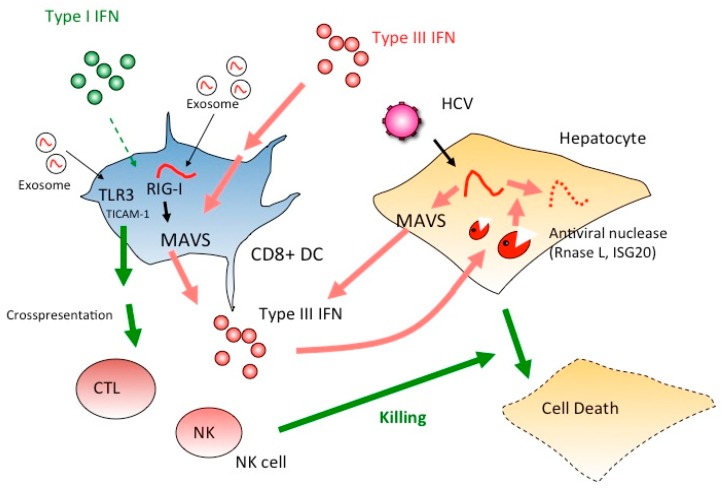
Type I IFN but not type III IFN mature DC to proliferate CTL and NK cells. If type III IFNs mainly act on DCs, CTL and NK cells cannot be activated as effectors for the elimination of HCV-infected hepatocytes. Instead, ISG20 and RNase L induced in hepatocytes by IFN-lambda participate in elimination viral RNA of HCV-infected cells. IFNLR is a receptor for IFN-lambda, but unlike IFNAR, this receptor cannot activate CTL and NK cells. Thus, ISG20 and RNaseL act as major effectors in hepatocytes to suppress viral infection via IFN-lambda. Once type I IFN is induced, TLR3 is up-regulated in DC and DC-mediated cellular immunity is evoked to kill virus-infected cells.

From these results, hepatocytes initially produce type III IFN in response to HCV RNA, and CD8+ DCs have a receptor for IFN-lambda which can be generated from hepatocytes with RNA stimulation (Figure 2). Notably, type III IFNs produced from hepatocytes in HCV infection trigger the signal for amplification of type III IFN production. Type III IFN further induces IFN-inducible genes, ISG20 and RNaseL in hepatocytes [61,62], which degrade HCV RNA in hepatocytes to eradicate HCV. In addition, type I IFN is produced in DCs to amplify type III IFN production. Cross-presentation and NK activation are characteristic of CD8+ DCs [60,62], but they appear not to be directly attributable to the produced IFN-lambda (Figure 2).

## 5. Extrinsically Added Hepatocyte Debris Containing HCV RNA Stimulates the TICAM-1 Pathway

In acute phase of HCV infection, HCV-RNA is sensed by hepatocyte RIG-I, and the MAVS pathway is activated in infected cells to induce type I and III IFNs. However, the study in primary-cultured hepatocytes, type I IFN induction is barely detected, while type III IFN induction is observed in an initial phase [30]. On the other hand, after cell death response occurs in an early HCV infection [10,63], exosomes and cell debris (where HCV-RNA fragments are contained) can act as PRR-stimulators. If the debris of necrotic HCV-infected hepatocytes are co-cultured with human monocyte-derived (Mo) DC *in vitro*, the MoDCs induce inflammatory cytokines and type I IFN, and mature into an active-staged DCs to induce CTL and NK activation [10]. This response is blocked in TLR3-knockdown MoDC, suggesting that TLR3 initiates the response to HCV-RNA in MoDC [10]. It is notable that the transcription factor IRF3 is a key to induce MoDC maturation to license NK/CTL activation [10]. Therefore, in the extrinsic pathway of HCV RNA recognition, IRF3 is phosphorylated through the TLR3/TICAM-1 pathway in MoDC, along with the production of type I IFN in DC, which facilitates CTL and NK induction against HCV. Simultaneously, type III IFNs are expected to be induced from human DCs. What happens in this complex situation in HCV infection is interesting, and need to be further confirmed. A convincing finding is that type III as well as type I IFN produced in HCV infection act on mouse CD8+ DCs, the process of which amplifies the production of IFN-lambda. The IFN-lambda then induces RNases (including ISG20 and RNaseL) in infected cells, resulting in the suppression of HCV replication [61,62].

## 6. TICAM-1 Signal Initiated by HCV RNA in Persistent Infection Induces Cellular Immunity

HCV infection becomes chronic in more than a half of patients with acute infection [64]. HCV persistent infection triggers fibrosis of the liver, further causing cirrhosis and hepatocellular carcinoma (HCC) [64,65]. In persistent infection, HCV NS3/4A protease inhibits RIG-I ubiquitination by proteolytic degradation of Riplet and MAVS by direct proteolysis [40,65,66]. This Riplet/MAVS proteolysis suppresses type I IFN production in the infected liver cells. Since NS3/4A continues to exert protease activity as long as HCV proteins are produced in the liver, IFN production is continuously suppressed in infectious foci. NS3/4A has several different subcellular localizations, including at the mitochondria, peroxisomes, ER and mitochondrial-associated ER membranes (MAM) [65,66]. MAM-localized NS3/4A would be a key role in regulation of IFN induction. The concept of “stress granule” [3,62] also fits the finding that MAVS is localized to MAM as with NS3/4A [40,66]. Hence, type I IFN production from infected liver cells is suppressed. Meanwhile, TLR3 is hardly expressed in normal hepatocytes, although its expression is induced in the endoplasmic reticulum of hepatocytes if hepatocytes are chronically infected, stimulated with polyI:C or converted to hepatoma cells [67]. But, serum levels of IFNs produced from the infected hepatocytes are minimal in patients. On the other hand, stromal cells including DCs induce type I IFN as described previously [10,52]. Cellular debris or exosomes induce maturation of DCs in a TLR3-dependent manner [10,68]. Type I IFNs as well as immune effectors NK/CTL can be induced along with TLR3 activation. This is reasonable since in a chronic infection status, immune activation via the RIG-I pathway is inhibited in infected host cells; the TLR3/TICAM-1 pathway may play an important role in immune activation via stromal cells and DCs [10,68].

HCV does not have a carcinogen in the virus particle that causes obvious malignant transformation in the virus particle, but core protein would act as a trigger for initial inflammation in local hepatocytes [69]. Additional viral factors may facilitate the formation of infectious foci with the aid of host innate response [6,8,70,71]. HCC occurs from the infectious foci accompanied with immune-modulation and inflammation. Hepatocyte-derived cell-debris and exosomes with RNA, may accelerate inflammation as well as host cell transformation that supplies the basis for regulation of carcinogenesis. Further studies are necessary to elucidate the relationship between pattern sensors and carcinogenesis in HCV.

## 7. Concluding Remarks

Here we show that multiple RNA sensors participate in HCV RNA recognition. HCV RNAs are sensed by RIG-I/MDA5 in infected hepatocytes, and type I and III IFNs are subtly liberated. HCV RNAs can be released out from infected cells as an alternative form such as exosome or debris. HCV RNAs outside the hepatocytes (in the form of exosome) are recognized by TLR3 in DCs, which evoke major cellular effectors as well as robust type I/III IFNs. These innate immune responses may participate in the regulation of pathogenesis of HCV in chronic hepatitis, cirrhosis and probably HCC.
